# A pilot study to evaluate the effect of Taeumjowi-tang on obesity in Korean adults: study protocol for a randomised, double-blind, placebo-controlled, multicentre trial

**DOI:** 10.1186/1745-6215-13-33

**Published:** 2012-04-07

**Authors:** Sunju Park, Jeong-Su Park, ChunHoo Cheon, Yong Joon Yang, Changsuk An, Bo-Hyoung Jang, Yun-Kyung Song, Hoyeon Go, Ju Ah Lee, Yongcheol Shin, Seong-Gyu Ko

**Affiliations:** 1 Center for Clinical Research and Genomics, College of Oriental Medicine and Institute of Oriental Medicine, Kyung Hee University, 1 Hoegi-dong, Seoul 130-701, Republic of Korea; 2 Department of Preventive Medicine, College of Oriental Medicine, Kyung Hee University, 1 Hoegi-dong, Seoul 130-701, Republic of Korea; 3 Department of Oriental Rehabilitation Medicine, College of Oriental Medicine, Kyung-won University, Seongnam 461-701, Republic of Korea; 4 Oriental Internal Medicine, Semyung University, Bongbang-dong, Chungju 390-711, Republic of Korea; 5 Korea Institute of Oriental Medicine, 1672 Yuseongdae-ro, Daejeon 305-811, Republic of Korea

**Keywords:** Taeumjowi-tang, obesity, efficacy, safety, randomised controlled trial

## Abstract

**Background:**

Obesity, which is described as excessive or abnormal body fat, increases the risk of diet-related diseases. In Korea and around the world, the prevalence of obesity has grown annually from 1998 to 2008. This growth has continued despite various therapeutic efforts. The discovery of new and alternative treatments for obesity should be considered an important priority. Taeumjowi-tang (TJ001), a traditional Korean medicinal extract consisting of eight herbs, is a widely used herbal remedy for obesity in Korea. However, the efficacy and safety of TJ001 have not been fully investigated in a clinical trial. The purpose of this pilot study is to estimate obesity-related parameters and to assess the efficacy and safety of TJ001.

**Methods:**

Our study is a randomised, double-blind, placebo-controlled, multicentre clinical trial of Taeumjowi-tang (TJ001). For this study, we will recruit obese Korean patients of both sexes, ages 18 to 65 years, from four university hospitals. A total of 104 subjects will be recruited. The participants will receive either 7 g of TJ001 or a placebo three times daily for 12 weeks. The primary end point will be the rate of subjects who lose at least 5% of their baseline body weight. The secondary end points will be changes in body weight, body mass index, waist circumference, hip circumference, waist/hip circumference ratio, lipid profiles, body fat composition, blood pressure, fasting glucose concentration, C-reactive protein and questionnaires related to the quality of life. The outcomes will be measured every 4 weeks. The study period will be 12 weeks and will include a total of five visits with each subject (at screening and at 0, 4, 8 and 12 weeks).

**Conclusions:**

The results of our study will inform various estimates of TJ001 and will serve as the basis for a larger-scale trial. This study will assess the efficacy and safety of TJ001 as an alternative herbal remedy for obesity.

**Trial registration:**

Current Controlled Trials ISRCTN87153759

## Background

The World Health Organisation (WHO) defines "obesity" as abnormal or excessive fat accumulation that may impair health [1]. Treatment of obesity is important because this chronic, noncommunicable disease causes not only a range of health problems, such as cardiovascular disease, various metabolic syndromes and certain cancers [1-3], but also social problems. In addition to the global incidence of obesity, obesity has become one of the most life-threatening problems in Korea. The Korea National Health and Nutrition Examination Survey showed that the overall prevalence of Korean adult obesity in 2008 was 30.7%, compared with 21.8% in 1998 [4]. The survey defined adults as individuals who are at least 20 years old, and obesity was defined as a body mass index (BMI) 25 kg/m^2 ^according to the definition of the International Association for the study of Obesity in the Western Pacific Region of the WHO [1,5].

Several nonpharmacological attempts to curb the increase in obesity, including dietary or exercise management, have been largely unsuccessful. These failures have encouraged specialists to develop treatments using pharmacotherapy [2]. Despite these therapeutic attempts, an efficacy and safety limit for conventional weight reduction therapies is apparent [6]. The demand for safe and effective anti-obesity agents is increasing, and herbal combinations are meeting those needs [7]. The relative importance and potential benefits of herbal preparations has extended their role as possible alternative methods for obesity therapies.

Taeumjowi-tang (TJ001; HANPOONG Pharm & Foods Co Ltd, Jeonju-si, South Korea) is a traditional Korean medicine preparation that originated from I Je-ma's Sasang constitutional medicine theory [8]. This theory is representative of the individualised medical approach, which is widely used to diagnose and treat disease in Korea. The Sasang typology explains specific disease susceptibility and drug response differences through distinctive pathology types. Taeumjowi-tang (TJ001) is a decoction consisting of eight herbal ingredients and is usually prescribed for Tae-Eum persons (Greater Yin person, or Tae-Eum-In) to regulate stomach-related symptoms such as jaundice, anhidrosis, stuffiness and the sensation of fullness [8-10]. Owing to the unique pathology of Tae-Eum, these individuals gain weight more easily than the other three constitutional types (Tae-Yang, So-Eum and So-Yang). The Sasang typological formulary for Tae-Eum persons contains many therapies related to obesity. Among the various preparations, TJ001 has become the treatment regimen for obesity most widely used by Korean medical professionals [11-15], and its use has been expanded to all types of obesity. Preclinical results have supported the antiobesity and hypolipidaemic effects of TJ001 [16-19]; however, the results of clinical studies have been insufficient [20,21]. Although many large, placebo-controlled trials of herbal combinations and dietary supplements showing antiobesity effects have been reported [22-27], large-scale trials of Taeumjowi-tang have not yet been conducted.

This study will be the first reported randomised, double-blind, placebo-controlled clinical trial of Taeumjowi-tang (TJ001) in obese adults in Korea. The main purpose of this study is to assess the efficacy and safety of medicinal herbal extract of Taeumjowi-tang through a 12-week randomised controlled trial (RCT) that produces obesity-related estimates.

## Methods

### Objectives and hypothesis

#### Objectives

The main objectives of this trial are to (1) evaluate the efficacy and safety of TJ001 in obese Korean adults, (2) explore estimates of obesity-related variables, including the amount of weight reduction, (3) discover the precise target population, (4) establish an appropriate primary end point and (5) estimate the proper treatment period.

#### Hypothesis

We hypothesise that the TJ001 responder rates with a 5% or greater weight reduction will be higher than those in the placebo group. We also expect that use of TJ001 will be relatively safe.

### Study design and period

**Figure 1:**
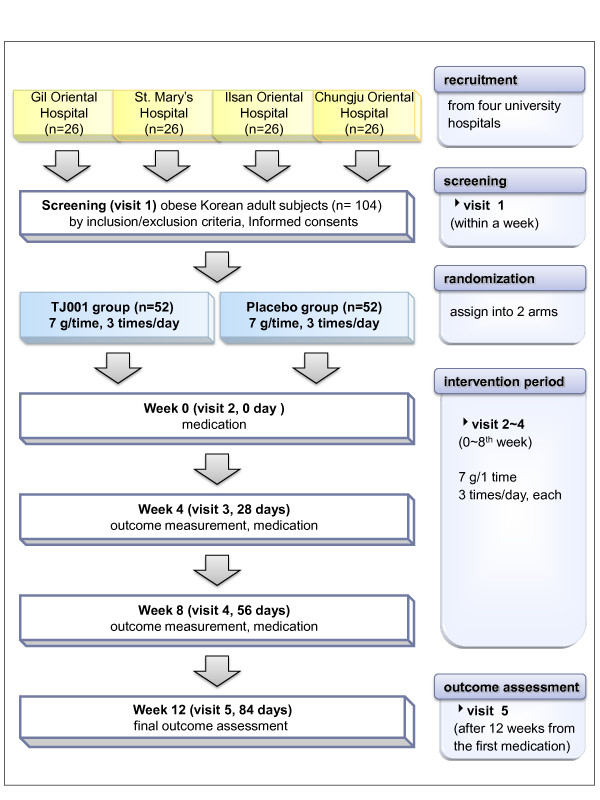
**Flowchart of the Taeumjowi-tang (TJ001) clinical trial**.

This study is a 12-week randomised, double-blind, placebo-controlled, multicentre trial at four tertiary university hospitals. Figure 1 shows the schematic flow of the study.

### Study groups

The study will include the following two arms: the TJ001 group (treatment arm) and the placebo group (control arm).

### Population

The subjects will be obese Koreans of both sexes and ages 18 to 65 years.

### Eligibility criteria

#### Inclusion and exclusion criteria

The inclusion and exclusion criteria are shown in Table 1.

**Table 1:** Inclusion and exclusion criteria^a^

Criteria
**Inclusion criteria**

1. Men and women ages 18 to 65 years
2. Individuals who meet one of the following criteria:
2.1. BMI ≥ 30 kg/m^2^
2.2. BMI 27 to 30 kg/m^2 ^with hypertension at a proper treatment- and blood pressure-controlled 95 to 145 mmHg
2.3. BMI 27 to 30 kg/m2 with non-insulin-dependent diabetes mellitus and fasting blood glucose < 7.8 mmol/L (140 mg/dl)
2.4. BMI 27 to 30 kg/m2 with hyperlipidaemia in a proper treatment regimen
2.5. BMI 27 to 30 kg/m2 and ≥ 236 mg/dl total cholesterol or ≥ 150 mg/dl triglycerides at screening
3. Agreed to low-calorie diet during the trial
4. Written informed consent for participation in the trial
5. Written informed consent for the genetic test
Exclusion criteria
1. Endocrine disease such as hypothyroidism or Cushing syndrome
2. Heart disease (heart failure, angina pectoris and/or myocardial infarction)
3. Uncontrolled hypertension (SBP > 145 mmHg or DBP > 95 mmHg)
4. Malignant tumour or lung disease
5. Cholelithiasis
6. Severe renal disability (sCr > 2.0 mg/dl)
7. Severe liver disability (2.5-fold the normal high range value for ALT, AST and ALP)
8. Non-insulin-dependent diabetes mellitus and fasting blood sugar 7.8 mmol/L (140 mg/d or over
9. Narrow-angle glaucoma
10. History or existence of neurological or psychological disease (schizophrenia, epilepsy, alcoholism, drug addiction, anorexia, bulimia and so on)
11. History of stroke or temporary ischaemic cardioplegia
12. History or existence of eating disorder such as anorexia nervosa or bulimia nervosa
13. Use of medication within the past 3 months that could have an effect on weight (appetite suppressant, laxative, oral steroid, thyroid hormone, amphetamine, cyproheptadine, phenothiazine or medication having an effect on absorption, metabolism and excretion)
14. Use of β-blocker or diuretic as hypertension medication within the past 3 months
15. Use of central nervous system medications or central nervous system stimulating weight reduction medications
16. Forbidden treatments (insulin, hypoglycaemic agent, antidepressant, antiserotonin agent, barbiturate, antipsychotic and concerns related to medication abuse)
17. Difficult-to-measure anthropometric dimensions due to anatomical changes such as resection surgery
18. History of weight reduction surgery, bariatric surgery and so on
19. Unable to follow instructions during the trial as judged by the investigator
20. Women who are pregnant, lactating, planning a pregnancy or women of child-bearing age who do not agree to proper contraception (birth control pill, hormone implant, IUD, spermicide, condom, abstinence and so on) (women of child-bearing age indicated to be within 2 years of menopause who did not receive hysterectomy, bilateral tubal ligation, bilateral oophorectomy, and so on)
21. Use of other investigational products within the past month
22. Weight reduction > 10% within the past 6 months
23. Cessation of smoking within past 3 months or an irregular smoking habit

^a^*ALP *alkaline phosphatase, *ALT *alanine aminotransferase, *AST*aspartate aminotransferase, *BMI *body mass index, *DBP *diastolic blood pressure, *IUD *intrauterine device; *SBP *systolic blood pressure, *sCr*, serum creatinine

#### Subject withdrawal criteria

The subjects who meet the criteria listed in Table 2 will be discontinued from treatment. The participants who will be withdrawn after randomisation will be followed for outcomes.

**Table 2:** Subject withdrawal criteria

Subject withdrawal criteria
1. Protocol violation: detection of eligibility violations, poor compliance (mean compliance < 70% at the last estimation) or noncompliance, use of any forbidden medication or treatment during the trial that could affect the study results, occurrence of other significant protocol violation during the trial
2. Occurrence of a serious adverse event
3. Subject has an acute reaction (allergy, shock and so on) to the investigational product
4. Detection of a systemic disease that was not discovered at the screening stage
5. Unable to progress because of worsening of preexisting disease
6. Subject's withdrawal of consent
7. Subject is uncooperative
8. Investigator's decision to terminate the process for the sake of the subject's health

### Interventions

Participating subjects assigned to either the treatment group or the placebo group will be instructed to take 7 g of TJ001 or placebo three times daily (a total of 21 g) for 12 weeks (84 days). TJ001 (Taeumjowi-tang granule extract, HANPOONG Pharm & Foods Co Ltd) is a compound of the powdered extract of several medicinal herbs, and the placebo will be similar to TJ001 in form, colour and odour. The components of TJ001 and the placebo are listed in Table 3.

**Table 3:** Constituents of interventions (TJ001 and placebo)^a^

	**TJ001 granule extract**^**b**^		
**Name of herb**	**Raw material code**	**Dose (g)**	**Content (%)**

*Semen Coicis*	M040487	3.75	NA
*Semen Castaneae*	M089646	3.75	NA
*Semen Raphani*	M210181	2.5	NA
*Schisandrae Fructus*	M040445	1.25	NA
*Liriopis tuber*	M040139	1.25	NA
*Herba Ephedrae*	M040135	1.25	NA
*Radix platycodi*	M040048	1.25	NA
*Acori Tatarinowii Rhizoma*	M051377	1.25	NA
	**Placebo**		
**Composition**	**Raw material code**	**Dose**	**Content (%)**
Lactose	NA	NA	87.99%
Starch	NA	NA	11.73%
Food colouring	NA	NA	0.28%

^a^*NA *not applicable. ^b^Korea Ministry for Health and Welfare classification 233 (ATC code A16AX).

### Lifestyle management

Dietary intake and the type and intensity of exercise will be recorded by the research coordinator at every visit but will not be managed strictly.

#### Dietary instructions

For dietary counselling, the subjects will be counselled to consume 20 to 25 kcal/kg. In other words, the participants will be asked to keep their dietary intake approximately at 1,500 kcal/day for men and 1,200 kcal/day for women. A dietary intake diary will be distributed to the subjects, and they will use a diary to calculate the number of calories consumed.

#### Exercise

As the trial will be conducted without lifestyle management, the participants will be allowed to maintain their usual exercise levels. However, intense, unusual workouts will not be permitted.

### Concomitant treatments and forbidden drugs

The subjects will be permitted to use medications only with the investigator's permission. The product name, use, dosage and duration of these medications will be recorded. The following concomitant treatments will be prohibited: central nervous system medications; central nervous system stimulating weight reduction medications; medications that could have an effect on weight (insulin, hypoglycaemic agents, oral steroids, thyroid hormones, cyproheptadine and phenothiazine); medications that affect absorption, metabolism or excretion; β-blockers and diuretics for hypertension treatment; amphetamines; antidepressants; antiserotonin agents; barbiturates; antipsychotics; and medicines that increase blood pressure or pulse, including decongestants that contain phenylpropanolamine, ephedrine or pseudoephedrine as well as medicines for coughs, common colds and allergies.

### Sample size calculation

A sample size of 104 subjects was estimated for 80% power at a significance level of 0.05 and an attrition rate of 20%. Participants will be selected according to the eligibility criteria.

No preliminary studies of TJ001 were available to aid in estimating the percentage of subjects with 5% or greater weight loss [21]. Therefore, we set the rate at 40% for the TJ001 group and 13% for the placebo group on the basis of a reference paper [28]. The formula for calculating the sample size when allocating subjects at a ratio of 1:1 (TJ001:placebo) is as follows:

$$\begin{array}{c} n=\frac{{\left\{z_{1-\alpha }\sqrt{2\bar{p}\left(1-\bar{p}\right)}+z_{1-\beta }\sqrt{p_{t}\left(1-p_{t}\right)+p_{c}\left(1-p_{c}\right)}\right\}}^{2}}{{\left(p_{t}-p_{c}\right)}^{2}}\\ \;=\frac{{\left\{1.96\sqrt{2\left(0.265\right)\left(0.735\right)}+0.84\sqrt{\left(0.40\right)\left(0.735\right)+\left(0.13\right)\left(0.87\right)}\right\}}^{2}}{{\left(0.40-0.13\right)}^{2}}\approx 41 \end{array}$$

p_t_

The 5% or greater responder rate of the initial weight in TJ001 group = 40%.

p_c_

The 5% or greater responder rate of the initial weight in the placebo group = 13%.

$$\bar{p}:\left(p_{t}+p_{c}\right)/2=26.5\%\;z_{1-\alpha /2}=1.96,\;z_{1-\beta }=0.84$$

Allowing for an attrition rate of 20%, the number of subjects in each group is 52.

$$n^{*}=\frac{n}{\left(1-0.2\right)}=51.25\approx 52$$

Therefore, a total of 104 subjects are needed for this trial.

### Randomisation method

The study subjects who satisfy the eligibility criteria will be randomised using a web-based randomisation program at an independent centre (Medical Research Collaborating Center of Seoul National University Hospital http://mrcc.snuh.org/). This program will be set to allocate participants equally to each site at a ratio of 1:1. Each of the four sites will be allocated 26 participants. The TJ001 and placebo groups will each be allocated 52 participants. The randomisation program will be designed with a four-patient block randomisation.

### Blinding

Both the investigator and the subject will be blinded regarding the assignment of the study drugs. The contract research organisation (CRO; Kyung Hee University, Center for Clinical Research and Genomics) of the sponsor will label the investigational drugs by the randomisation code number. The labelled experimental products will be provided to the trial sites by the CRO.

### Recruitment

Participants will be recruited through posted notes on the bulletin boards at four hospitals: Catholic University of Korea Seoul St Mary's Hospital (located in Seoul, South Korea), Dongguk University Ilsan Oriental Hospital, Kyungwon Gil Oriental Medical Hospital (Ilsan and Incheon, South Korea, both satellite cities near Seoul) and Semyung University Oriental Medicine Hospital (Chungju, a midsized city located in central South Korea).

### Study schedule

The measurements that need to be carried out at each visit are listed in Table 4.

**Table 4:** Study schedule of TJ001 clinical trial (12 weeks)^a^

	Initial screening b		Treatment period		
	
Measurement items	Visit 1 (-7 days)	Visit 2, week 0 (0 days)	Visit 3, 4 weeks (28 days)	Visit 4, 8 weeks (56 days)	Visit 5, 12 weeks (84 days)
Informed consent	Yes				
Demographic characteristics^c^	Yes				
Vital signs^d^	Yes	Yes	Yes	Yes	Yes
Medical/drug use history	Yes				
Smoking/drinking status	Yes		Yes	Yes	Yes
Physical examination^e^	Yes	Yes	Yes	Yes	Yes
Laboratory tests^f^	Yes				Yes
Thyroid hormones^g^	Yes				
Lipid test, CRP level^h^		Yes			Yes
Abdominal computed tomography for body fat		Yes			Yes
Blood sample for genetic test		Yes			
Electrocardiography	Yes				
Pregnancy test	Yes			Yes	Yes
Inclusion/exclusion criteria check		Yes			
Dietary intake measurement^i^		Yes	Yes	Yes	Yes
Concomitant medication		Yes	Yes	Yes	Yes
Adverse event			Yes	Yes	Yes
Questionnaires^j^		Yes			Yes
Questionnaire for Sasang					
Constitution Classification	Yes				
II					
Exterior Cold					
Disease induced from the		Yes	Yes	Yes	Yes
Esophagus affected by					
Cold' questionnaire in Tae-Eum-In persons Compliance calculation			Yes	Yes	Yes

^a^*CRP *C-reactive protein. ^b^The initial screening is performed within 1 week of the start of the study period. The visit windows for each participant are ± 3 days. ^c^Sex, date of birth, age, contact address and telephone number. ^d^Blood pressure (mmHg), pulse (beats/minute) and body temperature (°C). ^e^Body weight (kg), height (cm), waist circumference (cm) and hip circumference (cm). ^f^Blood tests are performed for haemoglobin, hematocrit, red blood cell count, white blood cell count, platelet count, total protein, albumin, total bilirubin, alanine transaminase, aspartate transaminase, alkaline γ-glutamyltransferase, uric acid, blood urea nitrogen/creatinine ratio, fasting plasma glucose, creatinine kinase and urine test for colour, specific gravity, pH, protein, glucose, ketone, urobilinogen, bilirubin, nitrite, red blood cells and white blood cells. ^g^Thyroid-stimulating hormone and free thyroxine are measured at the time of screening. ^h^Total cholesterol (mg/dl), high-density lipoprotein cholesterol (mg/dl), triglyceride (mg/dl) and CRP. ^i^Recorded in kilocalories using 24-hour dietary recall methods by a dietician or clinical research coordinator. ^j^Korean obesity-related quality of life (KOQOL) scale and Korean version of Eating Attitudes Test-26 (KEAT-26).

### Measurement tools

#### Anthropometric measuring for height, weight, waist circumference and hip circumference

All measurements will be taken by well-trained examiners using standard procedures. Measurements will be made while participants are dressed in light garments and with bare feet. Height and weight will be measured in the standing position to the nearest 0.1 cm and 0.1 kg, respectively. A measuring rod with a movable headpiece will be mounted on a balance beam scale. The waist circumference (WC), recorded to the nearest 0.1 cm, will be measured midway between the lower margin of the last rib and the top of the pelvic bone in a horizontal plane using plastic tape. The hip circumference (HC), recorded to the nearest 0.1 cm, will be measured at the horizontal level of the widest part of the hip. BMI is calculated by dividing weight in kilograms by height in square meters [29]. We will record the subjects' weight loss in kilograms at every visit and calculate BMI and the rate of monthly weight loss in both groups [30].

#### Questionnaires

##### Questionnaire for the Sasang Constitution Classification II

The Questionnaire for the Sasang Constitution Classification II Sasang Constitution Classification II classifies a person into one of four types of constitution according to personal traits based on I Je-ma's Sasang constitutional medicine theory [31]. We will use this questionnaire to classify obese Korean adults. Additionally, we will use this questionnaire to evaluate whether there is an association between each constitutional type and the effect of TJ001.

##### Exterior Cold Disease induced from the Oesophagus affected by Cold questionnaire in Tae-Eum persons

Exterior Cold Disease induced from the Esophagus affected by Cold questionnaire is used to evaluate symptoms that can occur in people of the Tae-Eum constitutional type according to the Sasang constitutional medicine theory [8]. We will apply this questionnaire to examine whether there is a relationship between the tendency toward obesity in Tae-Eum persons and their response to TJ001.

##### Compliance

All subjects will be asked to return the remaining investigational products at their next visit, and the rate of compliance (percentage) will be calculated on the basis of the returned products.

$$\text{Compliance}\left(\%\right)=\text{1}00-\text{returned}\;\text{products}/\text{expected}\;\text{intake}\times \text{1}00.$$

Investigational drugs will be distributed in packs of 100 at each visit.

100=3times/day×7days/week×4weeks/visit+16extras.

### Outcomes

Both primary and secondary end points will be measured at each visit according to the study schedule (Table 4).

#### Primary outcome

The primary outcome is the rate of subjects who have lost 5% or more of their baseline body weight [28,30,32].

#### Secondary outcomes

Both within-group and between-group analyses will be performed for each outcome. The differences in the following variables between the baseline (visit 1) and the last visit (visit 5) will be calculated.

• Body weight (kg)

• BMI (kg/m^2^)

• WC (cm)

• HC (cm)

• Waist/hip circumference ratio (WHR) change

Lipid profile: total cholesterol, triglyceride, HDL cholesterol, LDL cholesterol levels (mg/dl) (LDL cholesterol will be estimated using the Friedewald equation [33].)

Body fat composition: visceral fat area (cm^2^) and subcutaneous fat area (cm^2^) (Body composition will be evaluated by abdominal computed tomography.)

• Blood pressure (mmHg)

• Fasting plasma glucose concentration (mg/dl)

• C-reactive protein (mg/L) Questionnaires scores: Korean Obesity-related Quality of Life (KOQOL) and Korean version of Eating Attitudes Test-26 (KEAT-26)

#### Safety outcomes

All variables related to the safety assessment such as vital signs, general physical examinations, various test results (hematologic tests, biochemical tests, and urine tests) and adverse events (AEs) will be documented on the case report form (CRF) at every visit.

### Statistical analysis

#### Efficacy assessment

The primary outcome will be analysed using the intention-to-treat (ITT) method. The secondary outcomes will be assessed by both the ITT and the per-protocol (PP) methods. The full analysis set for ITT method will include all randomised subjects, regardless of their subsequent withdrawal after enrolment. The PP analysis will include patients who have completed the 12-week study term without any major protocol violations and have a compliance rate > 70%. Missing values will be replaced by using the last observation carried forward method. Continuous variables will be reported as means ± SD, and categorical variables will be reported as percentages. The baseline characteristics will be compared by either Student's *t*-test for continuous variables or the χ^2 ^test (Fisher's exact test when the expected value is < 5) for categorical data. Alternatively, McNemar's test will be used if the normality assumption is not satisfied for the continuous variables.

For the within-group analyses, primary and secondary outcome variables will be evaluated by using a paired *t*-test. Alternatively, for nonnormal distribution data, the Wilcoxon test will be performed. Analysis of covariance will be applied to analyse differences in each group at every visit, adjusting for age, baseline weight and BMI as covariates. Between-group comparisons for each variable will be performed using Student's *t*-test. Statistical significance will be defined as *P *< 0.05. PASW for Windows version 18.0 software (SPSS, Inc, Chicago, IL, USA) will be used for the analyses.

#### Safety assessment

Safety analyses will be performed for all subjects, who will be randomised and visited more than once after the initial screening. Safety-related variables will be analysed using the ITT method. Safety data will be stratified according to symptoms.

### Adverse event reporting

AEs will be recorded in medical diagnostic terminology. Detailed symptoms, duration, severity, causal relationships, actions taken, results and other information will be recorded for each AE. All AEs must be observed and recorded in the CRF in the AE report section. When an AE occurs, investigators should notify both the IRB and the regulatory authorities within 24 hours.

### Data quality control, data collection and data management

Data quality control will be achieved through monitoring during the trial. After checking the written CRF, well-trained clinical research associates of the CRO will collect the data. Data management will be performed by the CRO, Center for Clinical Research and Genomics, Seoul, Republic of Korea. All processes will be conducted using standard operating procedures.

### Ethical issues

This study has been approved by the institutional review boards (IRBs) at each of the four institutions: the IRB of the Catholic University of Korea Seoul St Mary's Hospital (reference KC09MNME0032), the IRB of the Dongguk University Ilsan Oriental Hospital (ref: SR-09), the IRB of the Semyung University Oriental Medicine Hospital (reference 2008-03) and the IRB of the Kyungwon Gil Oriental Medical Hospital (reference 08-101)). Written informed consent will be obtained from each individual prior to enrolment. Research will be performed in compliance with the Helsinki Declaration and with the Good Clinical Practice Guidelines.

## Discussion

By exploring estimated obesity-related variables, our aim in this pilot study is to determine the precise target population, the primary end point and the intervention period. Throughout the trial we will evaluate the efficacy and safety of Taeumjowi-tang. Korea has a dual medical treatment system, and herbal medicine assessments are under special regulations as described in the WHO legal status review of traditional medicine. For traditional Korean medicinal herbs, the Korea Food and Drug Administration rules specify that they are permitted to be produced [34,35] if herbal preparations are listed in the 11 classic traditional Korean and Chinese medicine books [36]. As these preparations are considered to have historical evidence of efficacy and safety, clinical or toxicological results can be exempted. Our intervention, Taeumjowi-tang, is described in one of these classic medical books, the *Donguisusebowon *[8]. Despite its long history and widespread usage, few Taeumjowi-tang trials have been published [21,27,37]. Therefore, our trial could be considered a pilot study, and the efficacy and safety of Taeumjowi-tang should be assessed in a RCT.

We encountered difficulties in deciding several issues. First, we had difficulty in selecting the target population. We considered using stratified randomisation, because weight reduction is affected by sex, age and physical baseline characteristics such as initial weight and BMI [3,38,39]. However, these various strata would decrease the statistical power. We first needed to determine the general patterns of Korean obesity. To maximise both the power and the results within given research budgets, we will include adults over age 18 years and both sexes. We will carry out stratified analyses with sex as a stratum. Other covariates, such as age, weight and BMI at the initial screening visit, will be adjusted.

The second question was what the primary end point would be. Among various outcomes [40,41], we set a 5% or greater weight loss responder rate as the primary outcome because 5% or greater weight loss is considered to be clinically significant [28,30,32]. As the amount of weight loss can vary due to initial weight, our study team concluded that the rate of the subjects who achieved a weight loss of 5% or more from their baseline weight would be reasonable compared to the absolute weight change. Other parameters, such as BMI, weight, body fat composition and WHR, were set as secondary outcomes.

Third, we had difficulty in determining the treatment duration. Though a short-term medication period for chronic treatment may not be sufficient to observe accurate long-term effects [22,42], we prioritised feasibility. We decided on a 12-week treatment for the trial after reflecting on the opinions of the clinicians' general experiences and considering both the attrition rate and the effectiveness of the preparation. Attrition rate will increase if the treatment duration is prolonged for more than 3 months. Additionally, according to data reported in the literature [21,27,32,43,44], the intervention period of weight reduction ranges from 1 day to 18 months for complementary therapy RCTs. Among complementary therapy RCTs, 8-week and 12-week trials are the most common [23,26,27,44].

## Conclusions

We will assess the efficacy and safety of TJ001 in this trial. On the basis of our research results, we will be able to determine an adequate target population, a valid primary end point and an adequate treatment duration for TJ001. These results will be used to guide a future large-scale study. In addition, we expect that the results regarding the relationship between Sasang typology and obesity will lead us closer to personalised medicine.

## Trial status

The trial was first designed in 2009, and the study began the same year. The study is ongoing, and subject recruitment has not been completed.

## Abbreviations

AE: Adverse event; ATC: Anatomical therapeutic chemical classification; BMI: Body mass index; CRF: Case report form; CRO: Contract research organisation; CRP: C-reactive protein; ECDEC: Exterior cold disease induced from the oesophagus affected by cold; HC: Hip circumference; HDL: High-density lipoprotein; IRB: Institutional Review Board; ITT: Intention to treat; KEAT-26: Korean version of Eating Attitudes Test-26; KOQOL: Korean Obesity-related Quality of Life; LDL: Low-density lipoprotein; PP: Per protocol; RCT: Randomised controlled trial; TJ001: Taeumjowi-tang; WC: waist circumference; WHO: World Health Organization; WHR: Waist/hip circumference ratio.

## Competing interests

The authors declare that they have no competing interests.

## Authors' contributions

SGK substantially contributed to the general idea and design of the study. SGK and YKS directed the overall project and take responsibility for the project. SJP, HYG, BHJ, YCS, CHC, JSP, YJY, CSA, JAL and YKS took part in designing the protocol. SJP, BHJ and HYG planned the data analysis. SJP drafted the manuscript. All authors read and consented to the publication of the manuscript.
